# Influences of Emotion on Driving Decisions at Different Risk Levels: An Eye Movement Study

**DOI:** 10.3389/fpsyg.2022.788712

**Published:** 2022-02-04

**Authors:** Xiaoying Zhang, Ruosong Chang, Xue Sui, Yutong Li

**Affiliations:** School of Psychology, Liaoning Normal University, Dalian, China

**Keywords:** driving decision, negative emotion, eye movement, level of risk, visual process

## Abstract

To explore the influences of traffic-related negative emotions on driving decisions, we induced drivers’ three emotions (neutral emotion, traffic-related negative emotion, and traffic-unrelated negative emotion) by videos, then the drivers were shown traffic pictures at different risk levels and made decisions about whether to slow down, while their eye movements were recorded. We found that traffic-related negative emotion influenced driving decisions. Compared with neutral emotion, traffic-related negative emotion led to an increase in the number of decelerations, and the higher the risk, the more the number of decelerations. The visual processing time of the risk area was shorter in the traffic-related negative emotional state than that in the neutral emotional state. The less time drivers spend looking at the risk area, the faster they make their driving decisions. The results suggest that traffic-related negative emotions lead drivers to make more conservative decisions. This study supports the rationality of using traffic accident materials to conduct safety education for drivers. This article also discussed the significance of traffic-related negative emotions to social security.

## Introduction

The number of motor vehicles increases rapidly, which leads to an increase in traffic accidents. Traffic accidents have become one of the leading causes of death and disability. It poses a great threat to social security. Fatalities in a road traffic accident are caused by a series of factors including people, vehicles, roads, and the environment (Wang et al., 2019). Previous studies have indicated that drivers’ characteristics and behaviors play an important role in predicting traffic accidents (Özkan et al., 2006), and improper driving behaviors are the main cause of accidents and violations (Mesken et al., 2002). Improper driving behavior not only relates to the driver’s life and safety, but also increases a lot of hidden dangers, and even endangers the society. Driver’s emotion, as one of the main psychological factors, is sometimes not controlled by cognition, and may spontaneously influence driving behaviors (Strongman, 2003; Šeibokaitė et al., 2017; Maldonado et al., 2020). Emotion is a short, easily affected, and rapidly changeable state of mind (Zimasa et al., 2016), which arises when something important to us is at stake and calls forth a coordinated set of behavioral, experiential, and physiological response tendencies that together influence how we respond to perceived challenges and opportunities (Gross, 2002).

In the process of driving, drivers are often affected by the events they experience, especially those related to negative emotion. The relationship between negative emotions and driving behavior has attracted the attention of researchers (Nesbit and Conger, 2012; Bogdan et al., 2016; Sullman et al., 2017). Jallais et al. (2014) found that sadness increased the localization error rate, and anger made participants slower to locate road elements. Kovacsova et al. (2016) found that anger, hostility, nervousness, and being upset were associated with aggressive driving. Zimasa et al. (2016) found that negative emotions had a detrimental effect on cognitive processes in general and on driving safety in particular. In sum, previous studies often focused on the influence of a specific negative emotion such as sadness, anger, and hostility on driving behaviors, but when driving attentively in busy traffic, the driver could hardly tell what the exact emotion at the moment. However, the question of our study can be answered by examining the influence of negative emotions on driving decisions. Therefore, instead of distinguishing the specific types of negative emotions, this study only induced negative emotions to explore the influence of traffic-related negative emotions on driving decisions. Emotion arises with clear reasons. The duration of emotion is usually short and the whole process of emotion from arising to fading could be easily traced (Hu et al., 2013). Different studies have used different methods to induce emotions (Eherenfreund-Hager et al., 2017; Steinhauser et al., 2018). In the present study, we induced drivers’ negative emotions by watching videos, which was a simple and effective emotion-induced method.

The relationship between eye movements and cognition had attracted researchers’ attention for a long time (Posner, 1980). Some researchers have used eye-movement datasets over still images to evaluate visual attention models (Borji and Itti, 2013). Visual attention is controlled by top-down and bottom-up processing (Orquin and Loose, 2013). So risks on the road and drivers’ emotions jointly act on drivers’ visual processing speed of risk. Besides, studies have found that the driving decision is also affected by different situations. With the increase of risk rating, both the frequency of blinks and total duration decreased significantly (Charlton et al., 2014). We infer that the related events leading to negative emotions play a more dominant role and are easier to capture attention. Therefore, drivers with traffic-related negative emotions may be more sensitive to traffic-related risks and have shorter visual processing time for traffic risks.

Drivers’ eye movements exhibit different fixation patterns for different driving tasks. Land and Lee (1994) found that when driving along a tortuous road, the driver paid more attention to the “tangent point” on the inside of each curve. Eye fixations may reflect the processing state of the driver. Lemonnier et al. (2014) asked participants to operate a driving simulator to a crossroad and decided to stop or not. They found different visual patterns in different decision-making phases: high visual exploration (larger saccade amplitudes and shorter fixation duration) for the differentiation phase (leading to a prior decision), and lower visual exploration (smaller saccades and longer fixations) for the consolidation phase (leading to a final decision). Markov models of eye movement behavior in simple situations could even be linearly combined to predict behavior in complex tasks (Liu et al., 1998). Different driving decision studies use different decision tasks (Calisir and Lehto, 2002; Alvaro et al., 2018). In Torres et al. (2017), participants were shown images of traffic situations and asked to make decisions on braking tasks. This kind of task was easier to understand and more related to real life. Referring to Torres, we used the decision task of whether to slow down in traffic scenarios at different risk levels to explore the influence of emotions on driving decisions. We infer that the shorter time for which drivers gaze at the risk area is related to the faster speed at which they make driving decisions.

In summary, we induced drivers’ emotions by videos and recorded drivers’ driving decisions, reaction time, and total fixation duration to investigate the driving-decision processing of whether to slow down when drivers watched traffic pictures at different risk levels. We proposed the following hypotheses: (1) Drivers with traffic-related negative emotional states make more deceleration decisions, namely, the higher the risk level, the more the number of deceleration decisions. (2) Drivers with traffic-related negative emotional states have faster visual processing on the risk scene. (3) The shorter time for which drivers gaze at the risk area is related to the faster speed at which they make driving decisions.

## Materials and Methods

We asked the participants to watch a video and fill in the emotional assessment scale. After a successful emotion induction, the participants were shown a group of pictures at three risk levels and asked to make driving decisions about whether to slow down according to the traffic risk in the picture. In our study, each participant must be induced three emotions and complete three decision-making procedures. The specific experimental methods are as follows.

### Participants

G-power 3.1.9.7 is used to estimate the sample size (α = 0.05, 1−β = 0.80), with an effect size *f* = 0.40, and calculated minimum sample size *N* = 24 (Faul et al., 2009). Thirty-six college students with driving licenses were selected to participate in this experiment. One participant with unqualified eye movement calibration was deleted, and 35 participants remained (14 males, χ^2^ = 1.4, *p* = 0.237). The average age of participants was 25.17 years old, and the standard deviation (*SD*) was 3.07. Their average mileage driven was 5427.74 km and average time to obtain the driver’s license was 41.66 months. All of the participants were right-handed, had the normal naked or corrected vision, and had no color blindness or color weakness. All of the participants have read and signed the Informed Consent Form and received appropriate remuneration after the experiment.

### Experimental Apparatus and Materials

Eyelink 1000 Plus desktop eye tracker was used to collect eye movement data at a sampling frequency of 1,000 Hz. The stimulation was presented on a 19-inch LCD with a resolution of 1280 × 1024 and a refresh rate of 60 Hz. A chin-rest and forehead-rest minimized head movements. The eyes of the participant were about 60 cm from the center of the screen.

We prepared about 900 min of dashcam videos taken from the driver’s perspective. During filming, vehicles were driving around the city center, and the speed was about 40 km/h, the weather conditions were good. The vehicle ran on the road safely without traffic accidents. We asked a driver with more than 10 years of driving experience to take 180 pictures from the video, and each picture contained a traffic risk.

Then we asked 20 college students with driver’s license to evaluate the risk in the picture on a scale of 1–7, 1 represented a very low risk, 4 represented a medium risk, and 7 represented a very high risk. The program for rating pictures was compiled and presented by E-prime 2.0. The average score of 20 students’ ratings of a picture was the risk level score of this picture. We finally selected 60 low-risk pictures and 60 high-risk pictures from 180 pictures according to the risk level scores. The average risk level score of 60 low-risk pictures was 2.64 ± 0.62, significantly lower than 4 (*p* < 0.001). The average risk level score of 60 high-risk pictures was 5.47 ± 0.52, significantly higher than 4 (*p* < 0.001).

To avoid participants’ fatigue or carelessness, we added the no-risk pictures. We captured 60 pictures without obstacles ahead of the road from the dashcam videos as no-risk pictures. Finally, we got a total of 180 experimental pictures, including 60 low-risk pictures, 60 high-risk pictures, and 60 no-risk pictures. The pictures were 1024 × 542 pixels.

We randomly divided high-risk pictures, low-risk pictures, and no-risk pictures into three groups, each group contained 20 high-risk pictures, 20 low-risk pictures, and 20 no-risk pictures. Variance analysis was performed on the risk level scores of the three groups of pictures, and the results showed that there was no significant difference in the three groups of pictures, *F*(2,117) = 0.002, *p* = 0.998. The risk description of the three groups of pictures was shown in Table 1.

**TABLE 1:** Description of risk scenarios in the three groups of pictures.

Picture group	Risk level	Risk type	Risk description	Number
1	Low-risk	Vehicle	Vehicles ahead turn or brake	2
			Vehicles on the left or right side turn or merge	7
		Pedestrian	Pedestrians ahead	2
			Pedestrians on the left or right side	9
	High-risk	Vehicle	Vehicles ahead turn or brake	2
			Vehicles on the left or right side turn or merge	8
		Pedestrian	Pedestrians ahead	6
			Pedestrians on the left or right side	4
2	Low-risk	Vehicle	Vehicles ahead turn or brake	5
			Vehicles on the left or right side turn or merge	5
		Pedestrian	Pedestrians ahead	2
			Pedestrians on the left or right side	8
	High-risk	Vehicle	Vehicles ahead turn or brake	8
			Vehicles on the left or right side turn or merge	2
		Pedestrian	Pedestrians ahead	8
			Pedestrians on the left or right side	2
3	Low-risk	Vehicle	Vehicles ahead turn or brake	5
			Vehicles on the left or right side turn or merge	5
		Pedestrian	Pedestrians ahead	3
			Pedestrians on the left or right side	7
	High-risk	Vehicle	Vehicles ahead turn or brake	6
			Vehicles on the left or right side turn or merge	4
		Pedestrian	Pedestrians ahead	7
			Pedestrians on the left or right side	3

*1, 2, and 3, respectively, represent the first group of pictures, the second group of pictures, and the third group of pictures.*

### Materials of Emotion Induction

There were three emotion induction videos in our study. The 4 min 40 s of the traffic accident scene and warning education clip video induced traffic-related negative emotion. The 6 min 20 s disaster film “Aftershock” induced traffic-unrelated negative emotion. And the 5 min scenery video induced neutral emotion.

The traffic accident scene and warning education clip video contained real traffic accident scenes and two traffic accidents caused by dangerous driving behaviors, which were performed by professional actors. Some earthquake scenes were taken and edited from the film named “Aftershock.” Traffic accidents and earthquakes are both dangerous events that exist in real life, bringing loss of life and property. We used the two videos to induce traffic-related negative emotion and traffic-unrelated negative emotion.

### Emotion Assessment

Three pairs of adjectives (unpleasant–pleasant, relaxed–tense, tired–vigorous) were used to measure emotion (Matthews et al., 1995). Referring to Hu et al. (2013), another dimension was added to rate overall feeling: feeling bad–feeling good. Scale endpoints were 1 and 5. Cronbach’s α of the scale is 0.89. The average score of the four items was used in the statistical analysis.

### Area of Interest

**FIGURE 1:**
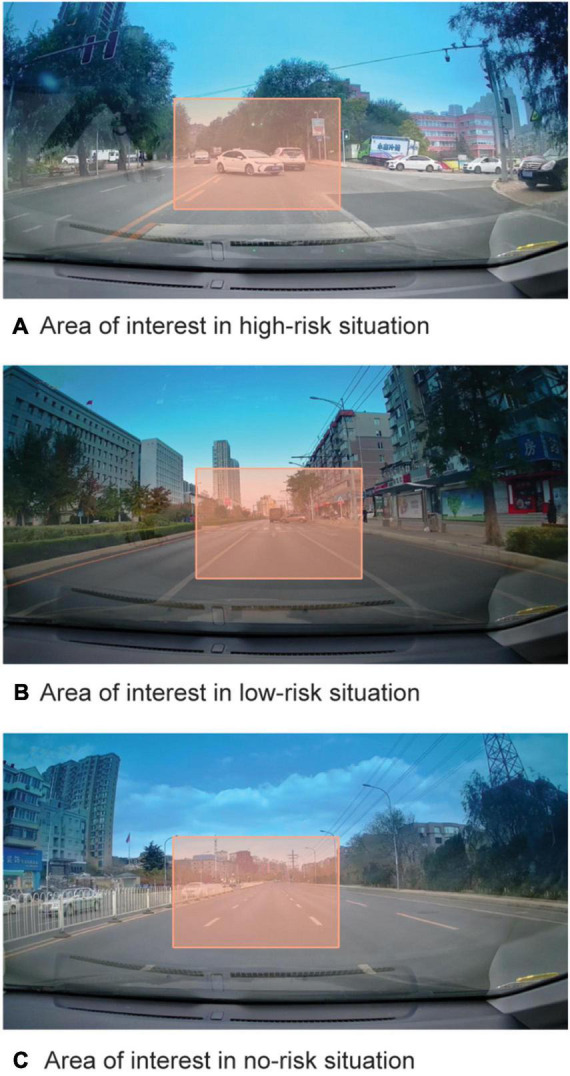
Areas of interest in different risk situations. **(A)** Area of interest in high-risk situation. **(B)** Area of interest in low-risk situation. **(C)** Area of interest in no-risk situation.

The eye movement data we analyzed were in the areas of interest. This study aimed to explore the influence of traffic-related negative emotion on deceleration driving decisions based on the risk level of the picture. Therefore, we took the risk area in the picture as the area of interest and focused on whether the driver’s visual processing of the risk area was related to the driving decision. So, areas of interest for high-risk and low-risk pictures were drawn where the traffic risk appeared. There was one risk in every picture, and the area of interest covered the risk. As the baseline condition, the area of interest of the no-risk picture was close to the area of interest of the risky picture, and the size was the same. The area of interest of each picture was 300 × 200 pixels (see Figure 1).

### Experimental Design

The study adopted a two-factor within-participant design of 3 (emotion type: neutral emotion, traffic-related negative emotion, and traffic-unrelated negative emotion) × 3 (risk level: high-risk, low-risk, and no-risk). The emotion type and risk level were within-subject variables. The dependent variables were reaction time, decision making and total fixation duration. The total fixation duration refers to the total time of all fixation points in the area of interest. The total fixation duration represents the total processing time of the area of interest.

### Experimental Sequence

Each participant was induced to experience three emotions (neutral emotion, traffic-related negative emotion, and traffic-unrelated negative emotion). To avoid the practice effect, we prepared three groups of pictures, each group contained 20 high-risk pictures, 20 low-risk pictures, and 20 no-risk pictures, which were presented in random order.

We used A, B, C to represent the three emotions and 1, 2, 3 to represent the three groups of pictures. We used permutation and combination to calculate the experimental sequences. The three emotion types form six sequences: A B C, A C B, B A C, B C A, C A B, and C B A. The three groups of pictures form six sequences: 1 2 3, 1 3 2, 2 1 3, 2 3 1, 3 1 2, and 3 2 1. We combined the sequences of emotion types with the sequences of three groups of pictures and then formed 36 experiment sequences. So, there were A_3_^3^ (three kinds of emotions) × A_3_^3^ (three groups of pictures) = 36 kinds of experiment sequences in Table 2. We randomly assigned participants to the experiment sequences.

**TABLE 2:** Arrangement and combination of emotion types and picture groups.

Emotion type	Picture group					
	1 2 3	1 3 2	2 1 3	2 3 1	3 1 2	3 2 1
A B C	A1 B2 C3	A1 B3 C2	A2 B1 C3	A2 B3 C1	A3 B1 C2	A3 B2 C1
A C B	A1 C2 B3	A1 C3 B2	A2 C1 B3	A2 C3 B1	A3 C1 B2	A3 C2 B1
B A C	B1 A2 C3	B1 A3 C2	B2 A1 C3	B2 A3 C1	B3 A1 C2	B3 A2 C1
B C A	B1 C2 A3	B1 C3 A2	B2 C1 A3	B2 C3 A1	B3 C1 A2	B3 C2 A1
C A B	C1 A2 B3	C1 A3 B2	C2 A1 B3	C2 A3 B1	C3 A1 B2	C3 A2 B1
C B A	C1 B2 A3	C1 B3 A2	C2 B1 A3	C2 B3 A1	C3 B1 A2	C3 B2 A1

*A represents neutral emotion, B represents traffic-related negative emotion, C represents traffic-unrelated negative emotion. 1, 2, and 3, respectively, represent the first group of pictures, the second group of pictures, and the third group of pictures.*

### Experimental Operation Steps

To make the effect of emotion induction undisturbed, a practice experiment was carried out before the first emotion induction. After reading the instruction, the participants practiced 10 trials, and the practice experimental procedure was the same as the formal experimental procedure. The practice experiment was compiled and presented by E-prime 2.0.

After the practice experiment, the participants were asked to fill in the emotional assessment scale before the emotion induction and then watch an emotion induction video. After that, they filled in the emotional assessment scale again and then completed an experimental procedure, the material of an experimental procedure consisting of a group of pictures. According to the previous studies that the effect of emotional induction could last for approximately 5–15 min (Bouhuys et al., 1995; Västfjäll, 2002; Green et al., 2003; Zimasa et al., 2016), we designed the experimental procedure that could be finished within 3 min in this study. After the experiment procedure, participants were asked to calculate simple math problems and then rested for 10 min to restore calm.

**FIGURE 2:**
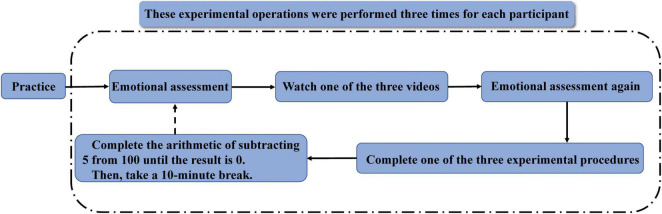
Experimental operation steps.

Then, the participants watched the second video and completed the second experiment procedure. Finally, the participants watched the third video and completed the third experiment procedure. Before each emotion induction, only the participants whose scores on the emotion rating scale were 2.5–3.5 could continue the experiment. If the participants’ emotional scores were not in this range, they were asked to continue to rest until their scores met the requirement. Each participant was randomly assigned to one of the 36 experiment sequences. The complete experimental operation steps are shown in Figure 2.

### Experimental Procedure

The experiment procedure was programmed and run by Experiment Builder, and the 9-point calibration method was used for eye calibration. The experimental procedure began after the calibration was successful.

The participant was asked to imagine that he or she was driving on the road. When faced with the traffic condition presented in the picture, if he or she wanted to slow down, press the “F” key; Otherwise, press the “J” key. They should make a decision as soon as possible.

**FIGURE 3:**
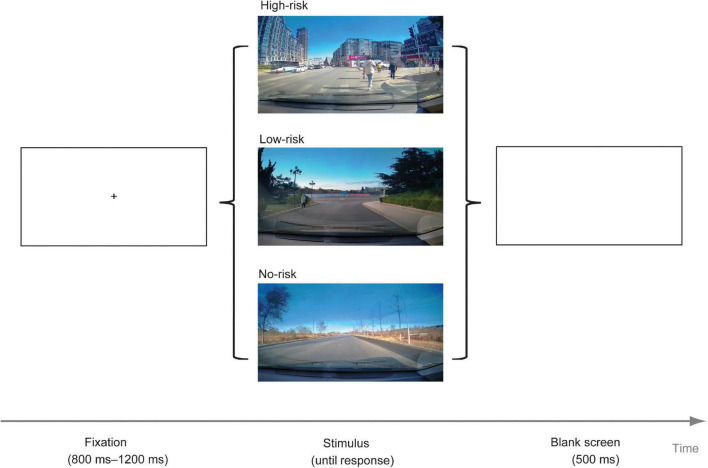
Flow chart of experiment.

We proposed the experimental procedure referring to the previous researches on driving decisions (Pammer and Blink, 2013; Pammer et al., 2018). First, the fixation point “ + ” was presented at the center of the screen at a random time between 800 and 1,200 ms. Then, the picture was presented until the participant made a decision. Finally, an empty screen of about 500 ms appeared (see Figure 3). We prepared three experimental procedures, and each procedure presented one of the three groups of pictures.

### Data Analysis

We used the Data Viewer of Eyelink 1000 Plus to export data. Trails with lost eye tracker data due to head movement, blinking, and other factors were eliminated. Data with a reaction time of more or less than 3 standard deviations were also eliminated. A total of 2.71% of the data was deleted.

The participants scored 1 point for choosing “F” and 0 point for choosing “J.” The deceleration decision score was the percentage of deceleration decisions in all trials under the same experimental condition. Reaction time and total fixation duration were measured in milliseconds.

We used SPSS 22.0 (SPSS Inc, 2000) to perform repeated measurement analysis of variance to investigate the effects of emotion induction. Then we used repeated-measures analysis of variance to investigate the effects of independent variables (emotion type and risk level) and their impact on participants’ deceleration decision score and reaction time. In addition, participants with different emotional states may have different driving decisions due to their visual processing patterns. Therefore, the next step was to use emotion type and risk level as independent variables and perform repeated measurement variance analysis using total fixation duration as a dependent variable to explore the differences in visual processing patterns for drivers in different emotional states when facing different risks levels. Finally, we conducted a correlation analysis of the deceleration decision score, reaction time, and total fixation duration of drivers in different emotional states when facing different risk levels to explore the relationship between driving decisions and visual processing patterns.

## Results

### Emotion Induction

**FIGURE 4:**
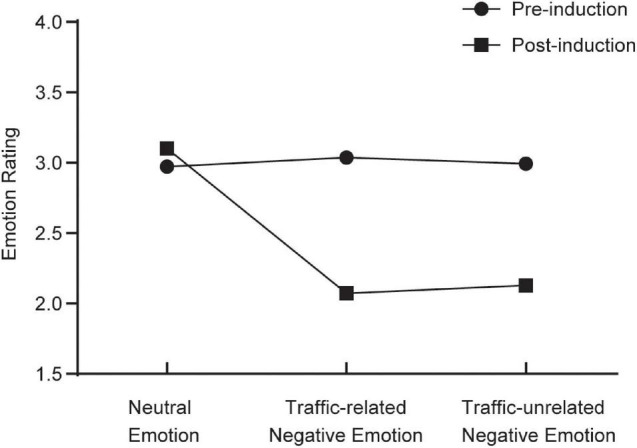
Pre-test and post-test emotions of the three groups.

We used repeated-measures analysis of variance to verify the effectiveness of emotion induction in this study. Before the emotion induction, there was no significant difference in emotion rating scores among the three groups, *F*(2,68) = 0.122, *p* = 0.885. After the emotion induction, the difference of emotion rating scores among the three groups was significant, *F*(2,68) = 154.387, *p* < 0.001, η^2^ = 0.820. The emotion rating score of the traffic-related negative emotion group was significantly lower than that of the neutral emotion group, *p* < 0.001, 95% CI = [−1.148, −0.909]. The emotion rating score of the traffic-unrelated negative emotion group was significantly lower than that of the neutral emotion group, *p* < 0.001, 95% CI = [−1.096, −0.847]. And there was no significant difference between the emotion rating scores of the two negative emotion groups, *p* = 0.458. These results indicated that emotion induction was effective (see Figure 4).

### Descriptive Statistics

This study explored the impact of drivers’ emotional states on driving decisions at different risk levels. Therefore, we proposed a 3 × 3 within-participant experimental design. To determine the effect of the independent variables on participants’ behaviors, we listed the descriptive statistics of the deceleration decision score and reaction time (see Table 3), which initially revealed that for different risk levels, participants’ driving decisions may be differentiated by their emotional states. To further explore the visual processing of the influence of participants’ emotional states on driving decisions, we listed the descriptive statistics of total fixation duration.

**TABLE 3:** Means and standard errors of reaction time, decision scores, and total fixation duration (*N* = 35).

Emotion type	Risk level					
	No-risk		Low-risk		High-risk	
	*M*	*SE*	*M*	*SE*	*M*	*SE*
	**Reaction time**					
Neutral emotion	1002	63	1263	64	944	52
Traffic-related negative emotion	988	58	1143	71	821	43
Traffic-unrelated negative emotion	974	62	1180	75	916	58
	**Decision score**					
Neutral emotion	0.07	0.01	0.64	0.04	0.97	0.01
Traffic-related negative emotion	0.10	0.02	0.73	0.04	0.97	0.01
Traffic-unrelated negative emotion	0.09	0.02	0.69	0.04	0.98	0.01
	**Total fixation duration**					
Neutral emotion	834	46	907	52	694	35
Traffic-related negative emotion	814	41	796	48	617	33
Traffic-unrelated negative emotion	762	45	813	55	661	41

*M, mean. SE, standard error.*

### Variance Analysis

**FIGURE 5:**
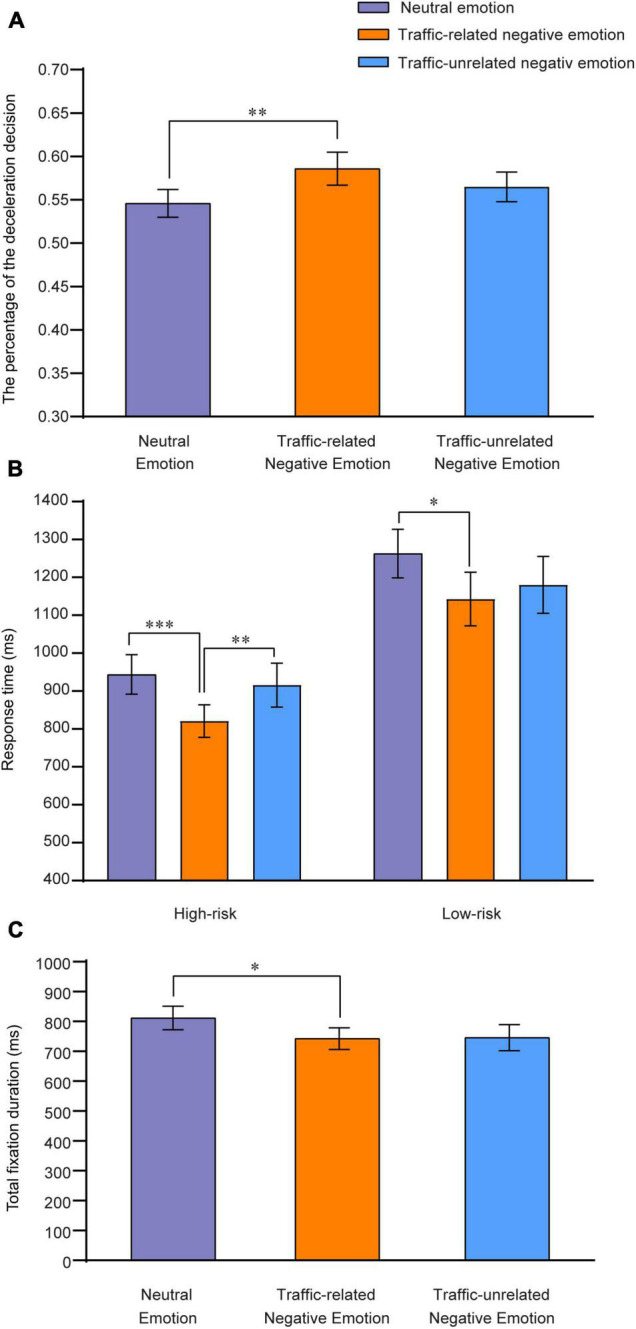
**(A)** Under the three emotional states, the percentage of deceleration decision in the total number of the trials under the same condition. **p < 0.01, Error bars represent the standard errors of the means. **(B)** At high-risk and low-risk levels, the reaction time of driving decision in three emotional states. *p < 0.05, ***p < 0.001. **(C)** The total fixation duration of driving decision in three emotional states.

We used emotion type and risk level as independent variables, and the deceleration decision score as dependent variable to perform a two-factor repeated measurement analysis of variance. The results showed that the main effect of emotion type was significant, *F*(2,68) = 3.91, *p* = 0.025, η^2^ = 0.103 (see Figure 5). The deceleration decision score in traffic-related negative emotional state was significantly higher than that in neutral emotional state, *p* = 0.018, 95% CI = [0.006, 0.078]. The main effect of risk level was significant, *F*(2,68) = 536.49, *p* < 0.001, η^2^ = 0.940. The deceleration decision score at high-risk level was significantly higher than that at low-risk level, *p* < 0.001, 95% CI = [0.201, 0.364], and no-risk level, *p* < 0.001, 95% CI = [0.844, 0.929]. The deceleration decision score at low-risk level was significantly higher than that at no-risk level, *p* < 0.001, 95% CI = [0.526, 0.682]. The interaction between the emotion type and risk level was not significant, *F*(4,136) = 2.202, *p* = 0.072, η^2^ = 0.061.

Emotion type and risk level were used as independent variables, and reaction time was used as a dependent variable to perform a repeated measurement analysis of variance. The results showed that the main effect of emotion type was significant, *F*(2,68) = 3.86, *p* = 0.026, η^2^ = 0.102 (see Figure 5). The reaction time of driving decision in the traffic-related negative emotional state was significantly shorter than that in a neutral emotional state, *p* = 0.021, 95% CI = [−161.039, −10.507]. The main effect of risk level was significant, *F*(2,68) = 35.10, *p* < 0.001, η^2^ = 0.508. The reaction time of driving decisions at the high-risk level was significantly shorter than that at the low-risk level, *p* < 0.001, 95% CI = [−379.346, −224.221]. The reaction time of driving decisions at the no-risk level was significantly shorter than that at the low-risk level, *p* < 0.001, 95% CI = [−308.667, −105.594]. The interaction between the emotion type and risk level was significant, *F*(4,136) = 3.06, *p* = 0.019, η^2^ = 0.082. A further simple effect analysis showed that the reaction time of traffic-related negative emotional state was significantly shorter than that of neutral emotional state at the low-risk level, *p* = 0.017, 95% CI = [−217.331, −23.059]. At the high-risk level, the reaction time of traffic-related negative emotional state was significantly shorter than that of neutral emotional state, *p* < 0.001, 95% CI = [−169.322, −76.692], and traffic-unrelated negative emotional state, *p* < 0.01, 95% CI = [−149.193, −40.784].

A repeated measurement analysis of variance was performed using emotion type and risk level as independent variables, and total fixation duration as dependent variables. The results showed that the main effect of emotion type was significant, *F*(2,68) = 3.54, *p* = 0.034, η^2^ = 0.094 (see Figure 5). The total fixation duration in traffic-related negative emotional state was significantly shorter than that in neutral emotional state, *p* = 0.046, 95% CI = [−137.815, −0.911]. The main effect of risk level was significant, *F*(2,68) = 28.95, *p* < 0.001, η^2^ = 0.460. The total fixation duration at high-risk level was significantly shorter than that at low-risk level, *p* < 0.001, 95% CI = [−236.840, −125.390], and no-risk level, *p* < 0.001, 95% CI = [−214.303, −77.420]. The interaction between the emotion type and risk level was not significant, *F*(4,136) = 2.262, *p* = 0.066, η^2^ = 0.062.

### Correlation Analysis of Variables

The above analysis explored the effect of drivers’ emotional states on driving decisions and visual processes at different risk levels. Since there was no risk in the no-risk pictures, we analyzed the relationship between visual processing and driving decisions in risky situations based on the above analysis results (see Table 4). Pearson correlation analysis was performed on the reaction time, deceleration decision score, and total fixation duration of different emotion types and risk levels. The results showed that the total fixation duration was significantly positively correlated with the reaction time in all conditions. The amount of time the drivers spent on the risk was related to the speed at which they made decisions.

**TABLE 4:** Correlation of reaction time, decision scores, and total fixation duration (*N* = 35).

Emotion type	Risk level		1	2	3
Neutral emotion	Low-risk	1. Total fixation duration	–		
		2. Reaction time	0.883**	–	
		3. Decision scores	0.210	0.124	–
	High-risk	1. Total fixation duration	–		
		2. Reaction time	0.826**	–	
		3. Decision scores	0.197	0.179	–
Traffic-related negative emotion	Low-risk	1. Total fixation duration	–		
		2. Reaction time	0.876**	–	
		3. Decision scores	0.121	0.017	–
	High-risk	1. Total fixation duration	–		
		2. Reaction time	0.785**	–	
		3. Decision scores	−0.045	0.079	–
Traffic-unrelated negative emotion	Low-risk	1. Total fixation duration	–		
		2. Reaction time	0.823**	–	
		3. Decision scores	0.100	0.065	–
	High-risk	1. Total fixation duration	–		
		2. Reaction time	0.846**	–	
		3. Decision scores	−0.076	−0.013	–

**p < 0.01.

## Discussion

According to the results of the analysis of variance with deceleration decision score, we found that drivers with traffic-related negative emotional states made more deceleration decisions, which may have been because they were more cautious about the traffic condition. The deceleration decision in this study involved a rapid matching of a perceptual representation to the stored knowledge in memory, which allowed drivers to identify traffic risks on the screen and determine how they should respond to them (Ratcliff et al., 2016). Hu et al. (2013) used a video clip depicting some cases of the traffic accident and tragic scenes after accidents to induce participants’ traffic-related negative emotion, and a video clip depicting cases of fire hazards and tragic scenes after accidents to induce traffic-unrelated negative emotion. They found that negative emotion significantly elevated drivers’ risk perception. However, the participants in the traffic-related group thought they were more likely to get involved with traffic accidents than those in the traffic-unrelated group. This may explain why drivers made more deceleration decisions. When faced with the decision of whether to slow down, drivers tended to give up the pursuit of driving speed to ensure safety. Furthermore, Charlton and Starkey (2016) suggested that drivers’ risk perception would affect their decision-making, especially in terms of speed. They found that when participants rated a higher risk level of road video from the driver’s perspective, they operated the driving simulator at a lower speed. The decision to slow down meant reducing the speed of driving, so drivers in this study made more deceleration decisions to avoid traffic accidents.

The results of the analysis of variance with reaction time showed that the speed of driving decisions at different risk levels was a significant difference. Compared with low-risk pictures, drivers made faster-driving decisions when faced with high-risk pictures. Perhaps this was because high levels of risk lead to a greater possibility of accidents, which caused drivers to be alert and make decisions more quickly. Low levels of risk, however, were less likely to lead to traffic accidents, and drivers might hesitate to make decisions, resulting in slower reaction time, even slower than no-risk level. The results further showed that the reaction time of traffic-related negative emotional state was significantly shorter than that of neutral emotional state at the low-risk level. The reaction time of traffic-related negative emotional states was significantly shorter than that of neutral emotional state and traffic-unrelated negative emotional state at the high-risk level. This may have been because the higher the risk level, the more likely drivers in the traffic-related negative emotional state were to involve themselves in traffic accidents. As a result, they made faster deceleration decisions to alleviate their own traffic-related negative emotional states and improve individual survival chances (Lang and Bradley, 2010). At the low-risk level, the driving decision under traffic-related negative emotional state was only significantly faster than that under neutral emotional state. With the increase of risk level, the driving decision under traffic-related negative emotional state was significantly faster than that under the other two emotional states.

According to the eye movements data results, we found that drivers in traffic-related negative emotional states had a shorter visual processing time of risk than that in a neutral emotional state. Previous studies have also shown that visual processing can distinguish the state and behavior of a driver. Underwood et al. (2003) found that experienced drivers and novice drivers have different visual attention patterns when driving on different types of roads (rural, suburban, and dual-carriageway). Walker and Trick (2019) found that emotional valence and arousal have different effects on drivers’ attention and driving performance (speed, steering, and hazard response). Attention was a selective process, which was usually conceptualized as being related to limited cognitive and brain resources, and there were severe limits on our capacity to process visual information (Carrasco, 2011). Drivers in the traffic-related negative emotional state were more worried about their involvement in the risk and more alert to risks. Therefore, they must consume the least cognitive resources as much as possible to make decisions as soon as possible to avoid possible traffic accidents.

Previous studies found that a driver’s intended actions can be inferred from their visual scanning behavior. Recognition performance could probably be significantly improved by improving the resolution of the gaze data so that features of the external visual scene could be identified (Liu and Salvucci, 2001). Results of the correlation showed that The shorter time for which drivers gazed at the risk area is related to the faster speed at which they made driving decisions. A dynamic driving simulator experiment found that the shorter the take-over time of drivers, the faster decision making and reactions, but generally worse in quality (Gold et al., 2013). In our study, there was no significant correlation between reaction time and deceleration decision score, we didn’t find the relationship between driving decision and reaction time.

In daily life, traffic management departments often organized examinees who were about to obtain driving licenses to watch traffic accident scenes and warning education videos, which immersed drivers in traffic-related negative emotional states. The result of this study showed that in this emotional state, drivers would have more conservative and safer driving behaviors, indicating that this kind of education and training method was effective, can promote drivers to drive carefully, and improve the level of traffic safety. The results of this study provide a scientific method and basis for drivers’ safety training: organizing drivers to watch the traffic accident scene and warning education clip video is an effective means of training. Correct and appropriate driving behavior of drivers is the premise to maintain driving safety, and road traffic safety is an important aspect of social security.

This study also has the following limitations. Firstly, previous studies showed that driving experience has an impact on different driving behaviors of drivers (Wallis and Horswill, 2007; Hills et al., 2018), this study did not consider this factor, so future research can explore the impact of negative emotions on experienced drivers and novices driving decisions. Secondly, this study focused on whether drivers’ deceleration decisions were affected by negative emotions at the moment when they faced the risk situations. Future research can explore whether drivers’ behavioral decisions are affected by negative emotions in the dynamic driving process, in order to improve the ecological validity of the experiment.

## Conclusion

This study confirmed that drivers with different emotional states had different driving decisions and visual processing when facing traffic conditions with different risk levels. Compared with neutral emotion, drivers in traffic-related negative emotional states made more deceleration decisions, and the higher the risk, the more deceleration decisions. The visual processing time of the risk area was shorter in the traffic-related negative emotional state than that in the neutral emotional state. The shorter time for which drivers gazed at the risk area is related to the faster speed at which they made driving decisions. The results of this study provide a scientific method and basis for driver safety training: organizing drivers to watch the traffic accident scene and warning education clip video is an effective means of training.

## Data Availability Statement

The raw data supporting the conclusions of this article will be made available by the authors, without undue reservation.

## Ethics Statement

The studies involving human participants were reviewed and approved by the Ethics Committee of Liaoning Normal University. The patients/participants provided their written informed consent to participate in this study.

## Author Contributions

YL and XZ conceived and designed the experiments. XZ and XS were involved in the data collection and analysis. XZ wrote the manuscript. YL and RC made critical comments and revised the manuscript. All authors contributed to the article and approved the submitted version.

## Conflict of Interest

The authors declare that the research was conducted in the absence of any commercial or financial relationships that could be construed as a potential conflict of interest.

## Publisher’s Note

All claims expressed in this article are solely those of the authors and do not necessarily represent those of their affiliated organizations, or those of the publisher, the editors and the reviewers. Any product that may be evaluated in this article, or claim that may be made by its manufacturer, is not guaranteed or endorsed by the publisher.
